# Mixed Features in Bipolar Disorder: assessing symptoms profiles and their relation with DSM-5 criteria

**DOI:** 10.1192/j.eurpsy.2023.1210

**Published:** 2023-07-19

**Authors:** R. Cafaro, M. Macellaro, B. Dell’Osso, T. Suppes

**Affiliations:** 1 Department of Biomedical and Clinical Sciences “Luigi Sacco”, University of Milan, Milan, Italy; 2 Department of Psychiatry and Behavioral Sciences, University of Stanford, Stanford, United States

## Abstract

**Introduction:**

Mixed states, the co-occurrence of manic and depressive symptoms, were recognized and described from the time of antiquity. DSM-5 first, and DSM-5-TR after, introduced the ‘’mixed features’’ specifier, defined by the presence of at least three non-overlapping opposite-pole symptoms during a syndromic depressive, hypomanic, or manic episode. Various manifestations, including irritability, distractibility, anxiety, psychomotor agitation, were excluded from the specifier, since they can occur during both depressive and hypo/manic episodes and other mental illnesses.

**Objectives:**

The objective of this study was to evaluate the phenomenology and prevalence of mixed states among bipolar disorder (BD) patients. We first assessed the frequency of specific features during different mood states. Then, we estimated the prevalence of mixed states by applying DSM-5 criteria, comparing it qualitatively with the one detected from psychometric questionnaires.

**Methods:**

In a naturalistic study, 903 adult outpatients with BD participating in the Stanley Foundation Bipolar Network were followed longitudinally across 14,213 visits for 7 years. The scores at the Inventory of Depressive Symptomatology–Clinician-Rated Version (IDS-C) and at the Young Mania Rating Scale (YMRS), administered at each visit, were used to define the mood episode and to assess the frequency of specific symptoms. In addition, we applied DSM-5 criteria for “with mixed features” to our sample, to examine a DSM-5-based construct.

**Results:**

Specific symptomatic profiles differentiate mixed states from pure ones (Figure 1 and 2). Mainly, a higher prevalence of irritability was found during mixed episodes, both depressive and hypo/manic, compared to pure depression (0.60 vs. 1.20, p < 0,001) and hypo/mania (0.82 vs. 1.54, p < 0,001), as reported at the 6th item of IDS-C.

Figure 1. Individual YMRS items scores in visits with pure depression, mixed depression, pure hypo/mania and mixed hypo/mania.

Figure 2. Individual IDS-C items scores in visits with pure depression, mixed depression, pure hypo/mania and mixed hypo/mania.

**Image:**

**Figure:**
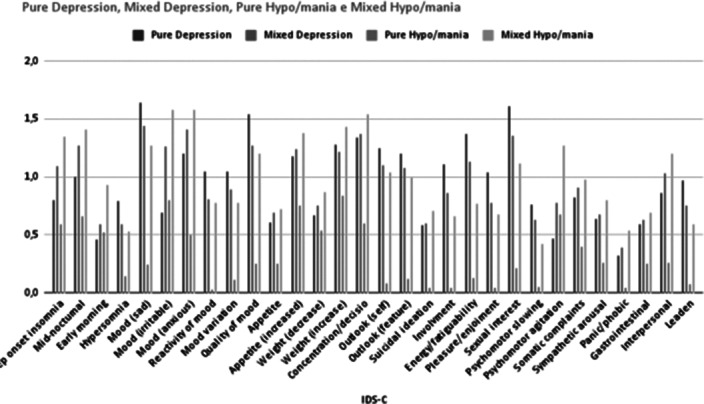

**Image 2:**

**Figure:**
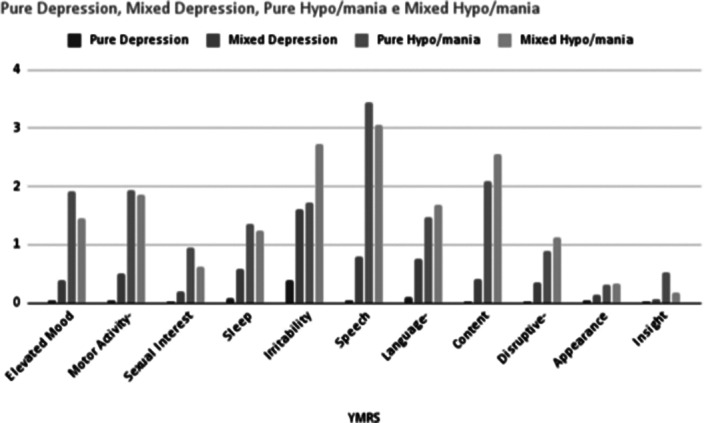

**Conclusions:**

Preliminary results of the present study showed that symptoms like irritability are strongly prevalent during mixed states. Moreover, the DSM-5 diagnostic criteria for “with mixed features” specifier for any of the mood episodes detected lower rates of mixed states, hence this criteria may yield inadequate sensitivity in recognizing patients suffering from such conditions.

**Disclosure of Interest:**

None Declared

